# Anti-COVID-19 Vaccination in the Italian General Population: Proactive Clinical Risk Analysis Using Failure Mode, Effects, and Criticality Analysis Technique

**DOI:** 10.3390/healthcare12242541

**Published:** 2024-12-16

**Authors:** Beatrice Balestracci, Giuseppe Candido, Lorenzo Federici, Chiara Parretti, Riccardo Tartaglia, Peter Lachman, Alessandra Bianco, Micaela La Regina

**Affiliations:** 1 Independent Researcher, 54023 Filattiera, Italy; beatricebale97@gmail.com; 2 Department of Engineering Sciences, Guglielmo Marconi University, 00193 Rome, Italy; g.candido@unimarconi.it (G.C.); c.parretti@unimarconi.it (C.P.); 3 Internal Medicine, Ligurian Health Authority n. 5, 19121 La Spezia, Italy; lorenzo.federici@asl5.liguria.it; 4 Department of Quality Improvement, Royal College of Physicians of Ireland, D02 X266 Dublin, Ireland; peterlachman@rcpi.ie; 5 Communications Department, Ligurian Health Authority n. 5, 19121 La Spezia, Italy; alessandra.bianco@asl5.liguria.it; 6 Clinical Governance and Risk Management, Ligurian Health Authority n. 5, 19121 La Spezia, Italy; micaela.laregina@asl5.liguria.it

**Keywords:** patient safety, proactive management, vaccination campaign, FMECA, SARS-CoV-2, COVID-19 pandemic, quality assurance

## Abstract

**Background**: Large-scale vaccination was crucial to address the global COVID-19 pandemic and its associated health risks, including fatal and disabling diseases. However, there were significant challenges to be overcome to ensure the safe and effective implementation of the vaccination program. The aim of the present study was to assess patient safety threats related to the anti-COVID-19 large-scale vaccination process. **Methods**: Between February and May 2021, we conducted a proper analysis to proactively identify risks and potential Failure Modes (FMs) in the COVID-19 vaccination process using the Failure Mode, Effects, and Criticality Analysis (FMECA) technique at an Italian Public Health Authority. A standardized risk scoring system was used to assess the severity, frequency, and detectability of events associated with potential failures. Criticalities were identified in both the preparatory and operational areas of the vaccination process, and several potential FMs were listed in descending order of risk score (Risk Priority Number, RPN) to ensure prioritization of interventions. **Results**: The most critical steps were found to be in the operational area rather than in the preparatory one. The highest RPNs were associated with failure or inadequate management of severe allergic reactions that can lead to serious harm and even death of the vaccinated person (RPN 60) and failure to keep updated vaccination teams’ knowledge (RPN 36). **Conclusions**: Ensuring patient safety and effective clinical risk management are crucial in mass vaccination campaigns. By prioritizing these aspects through collaboration with various stakeholders and implementing preventive measures, patient trust—on which vaccination campaign success relies—can be built and maintained.

## 1. Introduction

The 2019 SARS-CoV-2 pandemic (COVID-19) was a global health threat and public health emergency [1]. Various preventive measures were implemented, including the use of personal protective equipment, social distancing, hygiene practices, avoidance of crowded areas, contact tracing, rapid testing, improved indoor air quality, and last, but not least, mass vaccination [2,3]. Since January 2020, there has been an unprecedented global effort to develop safe and effective COVID-19 vaccines [4].

Vaccination against severe acute respiratory syndrome coronavirus 2 (SARS-CoV-2) was critical to control the pandemic and protect public health [5]. Authorized vaccines were shown to offer significant protection against severe COVID-19 cases, hospitalization, and death [6,7]. However, the process of developing a safe and effective vaccine is complex, and challenges may arise during its production, distribution, and administration, especially in developing countries where maintaining the cold chain requirements for the stability and efficacy of the molecule can be difficult [8]. 

The Italian Ministry of Health released a strategic plan for the COVID-19 vaccination campaign. This plan outlines the priority categories for vaccination and provides guidance on the procurement and disposal of vaccines [9]. The vaccination campaign in Italy progressed gradually, with a focus on first vaccinating vulnerable individuals and those who are ultra-vulnerable [10].

The vaccination plan was complex because of the vastness and diversity of aspects to be managed, along with the dynamic nature of the process and the constant updating of knowledge [11] on infection and available vaccines, along with greater information and education, positively influences the propensity for vaccination [12].

Standardization was critical to ensure the safety of such a complex process but may not be sufficient on its own. Then, proactive risk management is a fundamental strategy to this aim [13]. According to the U.S. Joint Commission, the Failure Mode and Effect Criticality Analysis (FMECA) methodology is useful for identifying and evaluating potential process failures and the severity of their effects [14]. FMECA is a decision-making tool that assigns a score to each risk, called Risk Priority Number (RPN), to prioritize risk management [15]. Originally developed by engineers to study complex systems, this method has been used to reduce risks in high-risk industrial processes [16,17]. This approach has been adopted successfully also in healthcare [14].

Here we present a study, conducted by a multidisciplinary working group coordinated by the Italian Network for Safety in Healthcare (INSH), an association committed to improving quality and safety in healthcare, with the support of a research team from the Department of Engineering Sciences at Guglielmo Marconi University in Rome. The aim was to assess the critical aspects of patient safety during the vaccination process, both in the preparatory and operational phases, and to propose targeted solutions. The study used the FMECA method to proactively assess clinical risk in the COVID-19 vaccine campaign to maximize safety and quality of clinical care.

## 2. Methods

A proactive assessment of the clinical risks associated with the anti-COVID-19 vaccination campaign at an Italian Public Health Authority was conducted using the FMECA technique.

FMECA is a systematic, stepwise method that proactively assesses what could go wrong (Failure Mode) and what the possible consequences could be (Effects Analysis) in a complex process and calculates the Risk Priority Number (RPN), which allows for prioritization of preventive measures.

The study followed the FMECA 5 steps: (1) assembling a multi-professional and multidisciplinary team; (2) mapping the process and activities; (3) brainstorming to identify potential failure modes in each activity, their effects and causes; (4) giving a numerical value (scoring) for the severity, frequency, and detectability of each failure mode and calculating the Risk Priority Number (RPN); and (5) suggesting corrective actions for selected Failure Modes [18,19].

A multidisciplinary and multi-professional research team was involved in predicting potential FMs that might arise during the anti-COVID-19 vaccination campaign. This analysis took place between February and May 2021 with several 120 min online brainstorming sessions with the project team. The sessions were facilitated by M.L.R.—qualified in clinical risk management and expert in risk—and included participants such as other Clinical Risk Managers (S.M. and M.U.), Psychologists (G.L and S.B.), a Pharmacist (A.I), a General Practitioner (A.S.), a Nurse (C.S.F.), and a Psychiatric Rehabilitation Technician (B.B.). Brainstorming rules were explained during the first session to participants. To simplify the collection and organization of information, word-processing programs were used during the meetings. Additionally, spreadsheets were utilized to effectively organize and visually present the data through tables.

**Figure 1:**
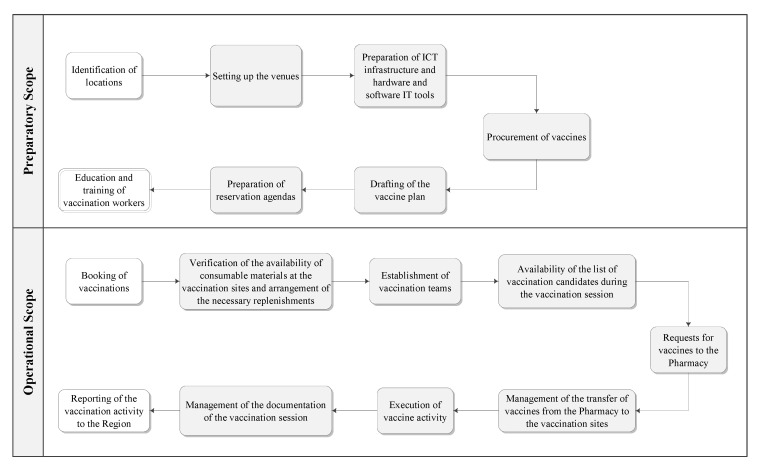
Phases of the anti-COVID-19 vaccine process: (1) preparatory scope and (2) operational scope.

At first, a flow chart was created to represent each stage of the vaccination process, divided into phases: preparatory and operational (see Figure 1). A list of all potential Failure Modes (FMs) was developed for each stage of the vaccination process by brainstorming. A scoring system including severity (S), occurrence (O), and detectability (D) of each effect was used to quantify the risks. Effects were divided among system and individual effects depending on the target of the failure. At each potential failure was assigned an RPN, calculated by multiplying the score assigned for severity, occurrence, and detectability score (RPN = S × O × D). The scores ranged from 1 to 5 (see Supplementary Materials File S1). To address the identified risks, corrective actions and barriers were defined and implemented [15]. The vaccination points included 5 hubs for adults and 1 hub for children. Three mobile teams provided vaccinations at nursing homes and residences for elderly and fragile people. The coexistence of different administration lines—different vaccines with different indications and contraindications administered at the same point—increased the risk of error. The follow-up on implemented measures was carried out by periodic on-site visits and collection of incident reporting. Three different validity tests were conducted to assess the effectiveness of the FMECA method: (I) face validity, (II) content validity, and (III) criterion validity. Face validity was positive in that the processes mapped by participants matched the observation of the work performed. However, other health professionals identified potential shortcomings that the FMECA teams had not detected. Content validity was assessed by presenting the FMECA results to other health professionals, and information was not provided on the results of this assessment. Finally, criterion validity confirmed the accuracy of FMECA, as the FMECA teams included all failures reported in the trust’s incident database.

## 3. Results

**Figure 2:**
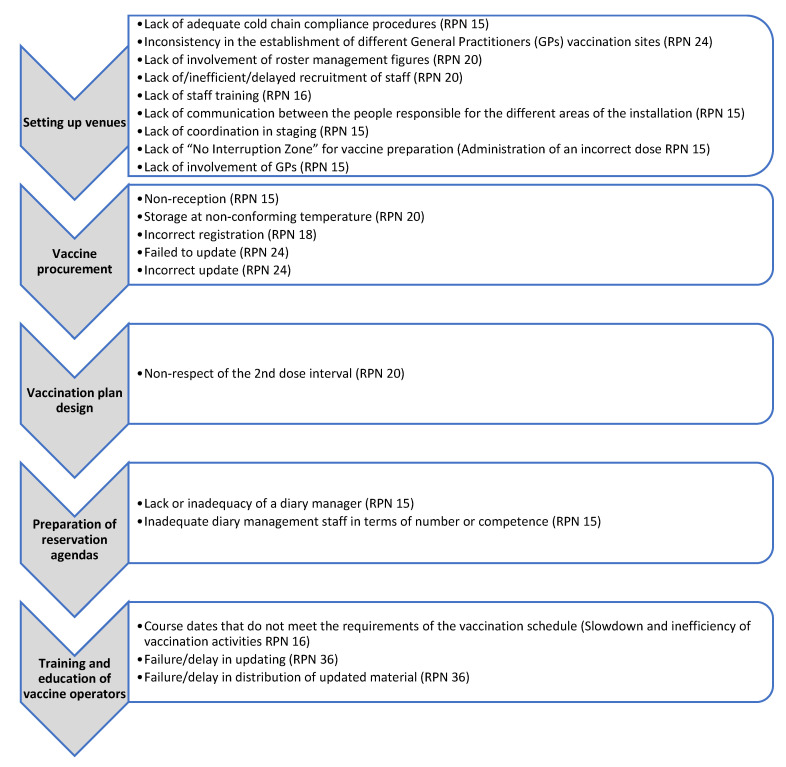
FMECA worksheet: possible failure modes in the preparatory scope.

**Figure 3:**
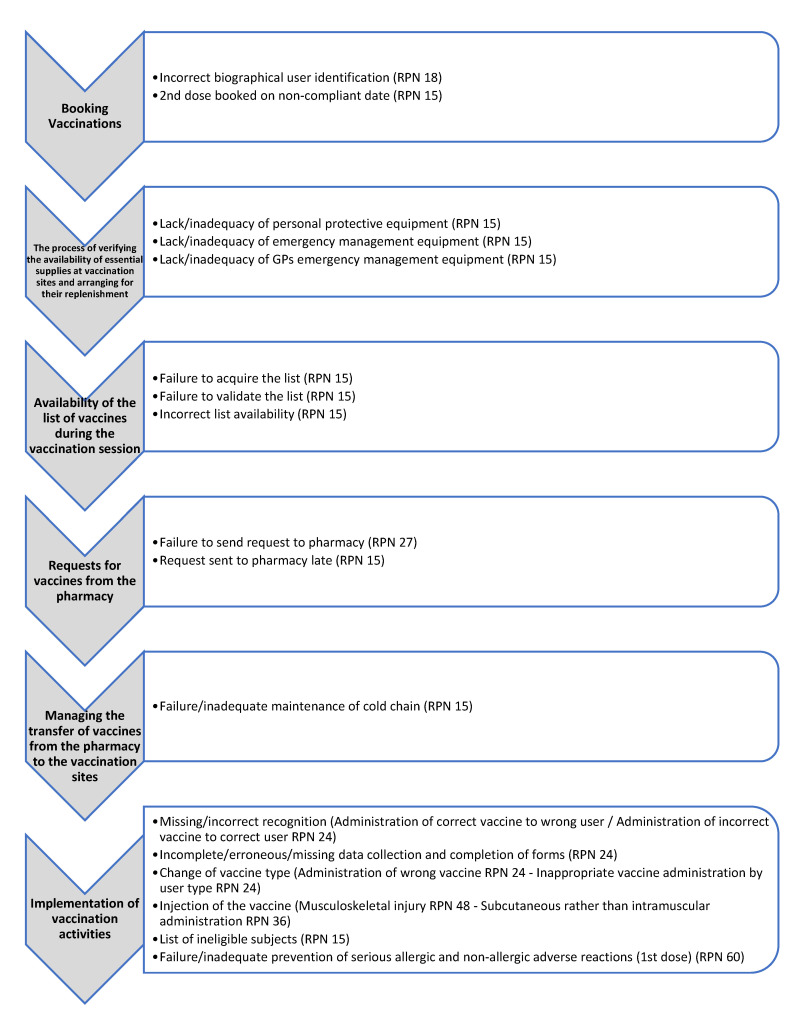
FMECA worksheet: possible failure modes in the operational scope.

A total of 157,792 doses of the COVID-19 vaccine were administered at the vaccination points and by the mobile teams at the study center until May 2021. The FMECA analysis identified two areas within the whole process: the preparatory and the operational areas. In the preparatory area, the research team identified 7 phases and 24 activities, with a total of 54 potential FMs. Preparatory phases included activities such as site identification and set-up, information and communications technology (ICT) configuration, vaccine procurement, vaccination plan writing, preparation of booking agendas, and training of vaccination teams. In the operational phase, which consisted of 9 steps and 19 predetermined activities, an additional 54 potential FMs were identified. Operational steps included activities such as management of vaccination bookings, checking supply availability, establishing vaccination teams, creating vaccine lists, making vaccine requests to the pharmacy, managing vaccine transfer, performing vaccinations, documenting the vaccination session, and reporting the activity to healthcare regional authorities. The most significant RPNs (≥15) assigned by the research group are reported in Figure 2 and Figure 3 (to view the full results, see Supplementary Materials File S1).

The focus of the study was on critical phases that not only had high scores but also had the potential to cause serious harm to patients or result in inefficient vaccination. The research team did not prioritize FMs based on conventional RPN values, as they believed that all FMs should be addressed regardless of their urgency. The team analyzed the RPN for each FM and determined whether the risk was acceptable or if improvements were necessary. FMs with the highest scores (RPN ≥ 15) were deemed high-risk and identified as priority areas for improvement. They were 20/54 in the preparatory area and 17/54 in the operational ones. Comparing the RPN scores registered in the two areas, the scores were generally higher in the operational one, maybe due to a major proximity to the user. The most critical phase in the preparatory area was the training and updating of vaccination teams’ knowledge (RPN 36), especially considering that anti-COVID-19 vaccines had never been administered before and were in continuous development.

In the operational area, the riskiest phases were as follows: (1) requesting vaccines from the pharmacy (RPN up to 27) and (2) administering the vaccine (RPN from 15 to 60). Failure or delay in sending requests to the pharmacy, or ordering inadequate quantities, may have delayed or canceled the entire vaccination sessions, slowing down the vaccination campaign and achieving the weekly/monthly targets in terms of vaccinated population, if frequently repeated. Administration errors, such as administering a vaccine to patients for whom it is not indicated, administering a second dose of a different vaccine in the presence of the principle of non-interchangeability (RPN 24), injection-related problems (RPN 36-48), and incorrect or incomplete medical history taking (RPN 24) can lead to increased adverse reactions. Above all failure/inadequate prevention of severe adverse reactions—allergic and not allergic ones—was recognized as the main risk to be addressed (RPN 60).

Based on the analyses carried out to mitigate the effects of identified adverse events, a series of 20 interventions were undertaken to provide operators with tools to keep the vaccination process under control. To ensure the effective organization of vaccination sites, several actions were taken. First, a “No Interruption Zone” was established to safely prepare vaccines. In addition, a person was designated to be responsible for the integrated management of staff recruitment and shifts, with the aim of recruiting the necessary staff in advance. This manager was also included in the vaccine task force to stay up-to-date on the vaccine plan. A local booking center was established to handle secondary needs such as re-scheduling vaccinations in case of necessary preliminary allergy consultation. Responsible persons were identified to monitor and procure vaccines, protective devices, and vaccination-reporting forms. In addition, refrigerators and data loggers were provided for proper storage of vaccines. The connectivity and ICT equipment of the vaccination sites were checked and set up, and emergency tools and procedures for electrical or telephone failures were planned. Finally, protocols were established for vaccine preparation and handling, requesting vaccines from the pharmacy, handling health emergencies, waste management, and absentee and reserve management. Several safety measures were implemented in the vaccine administration process. These measures included the following: verifying the identity of recipients by double identification technique, taking a complete medical history to detect any allergy, and ensuring that the vaccine was suitable for the intended target group. Color coding systems were used to differentiate the different types of vaccines. It was also suggested that the dose number and vaccine type be incorporated into the list of reservations. Different forms were recommended for the first and second doses of the vaccine, and appropriate administrative records were recommended for safety reasons (verification of dose number, vaccine type, and patient age against the licensed interval). In addition, it was suggested that longer needles be used for severely obese patients. Recommendations provisions from national, international, and local healthcare institutions along with alert reports about local lessons learned were promptly disseminated by WhatsApp Messenger^®^ 2.24.25.72 (vaccination team group), e-mail (vaccination team mailing list), and by a paper copy of the safety recommendations at any vaccination point, in order to prevent adverse events’ occurrence or re-occurrence. 

During the study period, 17 adverse events were reported by healthcare workers. These events included 13 cases where inappropriate vaccine doses were given, either due to the age or indication of the vaccine recipient. There was also one case of a large hematoma in a patient without coagulopathy, one case of a severe infection at the injection site, and two cases of person exchanges. Safety strategies were recommended to mitigate these risks. The results of FMECA were applied to the vaccination campaign to ensure the safety and effectiveness of the vaccination procedure.

## 4. Discussion

The effectiveness and safety of vaccination are achieved through a multi-step process involving various professional figures. In many Western countries, detailed recommendations are provided not only regarding medical indications for various vaccinations but also concerning structural and procedural aspects of the vaccination process. Although millions of vaccinations are performed each year, relatively few studies address the safety challenges posed by their management [20,21,22]. The safety of the COVID-19 vaccination campaign represented an even greater challenge as it was a global vaccination effort utilizing previously unknown types of vaccines (e.g., mRNA vaccines) [23]. The FMECA allowing to break down a process and pro-actively analyze single activities, highlighting the weakest steps in terms of reliability and/or the most critical ones in terms of risk, and understanding the nature and extent of the effects associated [18] is an excellent tool to make a critical process such as a mass vaccination in response to a pandemic safer for individuals and the community.

Above all, our study emphasizes as key outcomes the need to make efforts to prevent severe allergic and non-allergic reactions and to train and update vaccine administration teams to ensure individual patient safety, as well as improve supply logistics and timely operation of vaccination points to ensure community safety. In fact, delays in vaccine delivery and lack of staff responsible for managing shifts and recruitment lead to cancellation or delay of scheduled vaccinations.

Proper administration of vaccines is also essential to increase the number of immunized individuals. Errors in vaccine administration pose the highest risk, with severe allergic reactions being the most concerning as already reported in the literature [24]. These errors include administering a vaccine to patients for whom the vaccine is not indicated, administering a second dose of a different vaccine instead of the first, and confusion between the scheduling of the first and second doses. Additionally, incorrect or incomplete medical history information can lead to increased adverse reactions. These errors are often the result of communication and documentation problems, as well as insufficient training of health care providers and problems in the overall organization as also reported by Tussardi et al. [25].

Another issue is the inadequate storage of vaccines, which can result in the loss of doses and dose potency. There are reports in the literature of vaccine stocks not being accurately recorded in the pharmaceutical warehouse, resulting in discrepancies in immunization data. It is critical that national vaccination programs consider local storage conditions to ensure the smooth implementation of vaccination activities. This includes transportation and storage of vaccines in hospital facilities and pharmacies, as well as assessment of the condition of COVID-19 vaccination distribution centers. In addition, it is important to provide information on the temperature-based preparation and stability of vaccines to all healthcare workers who handle them [26]. Our analysis identified potential issues arising from the poor organization of vaccination sites, such as the lack of emergency equipment to manage anaphylactic reactions in GP offices [27]. Thus, the inclusion of general practitioners and community pediatricians in vaccine training is recommended. Other risks include misadministration, increased infections from improper handling of vaccine containers, not aseptic injection, and ambiguity in administration instructions. In our experience, this last aspect was prioritized from the start of the campaign, so that GPs and community pediatricians were included in the vaccination points and did not administer the vaccine in their offices. 

FMECA analysis enabled the clinical risk management unit to coordinate the implementation of risk control measures [28]. All good practices or recommendations [29] were disseminated to immunization staff including physicians, nurses, and administrators via e-mail and instant messaging platforms such as WhatsApp Messenger^®^. Updated support material such as operating instructions, vademecums, and reporting forms were also made available in hard copy at each vaccination point. In hubs where multiple vaccines were administered, a color-coded system was used to identify the route of each vaccine.

The FMECA analysis and the central role recognized by our organization to the risk management unit allowed us to proactively implement risk control measures [28]. Among the most impactful interventions was the continuous and priority attention to ensure effective and timely communication. All good practices or emerging recommendations [29] were promptly disseminated (7/7) to all vaccination personnel, including doctors, nurses, and administrators, via email and instant messaging platforms, such as WhatsApp Messenger^®^. Up-to-date support materials such as operating instructions, manuals, and reporting forms were also made available in hard copy at each vaccination point. In hubs where multiple vaccines were administered, a color-coding system was used to identify the route of each vaccine. In practice, this work allowed our organization to conduct a mass vaccination campaign with a limited number of adverse events. In fact, through the analysis, it was possible to identify risk containment solutions through the development of solutions before the event occurred, with a spin-off in terms of efficacy, safety, and coverage of the population. The results of the proposed activity can be compared with those of other studies conducted in Italy in the same period, which confirm the effectiveness of the approach in a highly complex system such as mass vaccination and which, as in the present work, confirm the complexity of the management process in particular of the logistical management aspects [11].

Of course, this study confirms not only the advantages but also the limitations of the FMECA methodology, already known and highlighted in the literature [30,31,32]. First, although the RPN tries to objectify the FMECA assessments, they are qualitative and the results are strictly linked to the experience of the professionals involved in the analysis and therefore may vary depending on the people conducting the analysis. Second, it would have been ideal to incorporate more input from patients and their families during the process mapping phase, but organizing such meetings is burdensome and also complex during a pandemic. Third, the present study refers specifically to the context of the COVID-19 pandemic. For a generalization of the results, comparisons with similar organizational scenarios in vaccination processes are needed. Finally, there is a lack of comparison with organizations that have not carried out proactive risk management or with others that have used reactive analysis tools based on clinical documentation and learning and reporting systems [30,31,32].

## 5. Conclusions

Clinical risk management serves as a crucial strategic resource. Adopting preventive strategies in highly complex processes, such as mass vaccination, not only ensures high safety standards for patients but also equips providers to anticipate and manage predictable adverse events effectively. By prioritizing patient safety through collaborative efforts with various stakeholders and implementing proactive measures, healthcare systems can foster patient trust—a cornerstone for the success of vaccination campaigns.

The study’s findings suggest several avenues for future research: I. Exploring the link between vaccination system organization and challenges: Methods like FMECA (Failure Modes, Effects, and Criticality Analysis) or others can evaluate the capacity of healthcare systems to address the demands of vaccination campaigns, particularly for new vaccines. II. Identifying high-risk individuals: This would facilitate the prevention of adverse events and support the development of personalized vaccination strategies, improving outcomes for vulnerable groups. III. Addressing equity and accessibility issues: Research could focus on ensuring fair access to vaccines for diverse social and professional groups during emergencies, while upholding ethical principles. IV. Optimizing the role of general practitioners and pharmacists: Investigating the organization and management of these professionals could enhance their contributions to vaccination campaigns, ensuring more effective delivery and monitoring of vaccines.

## Data Availability

All data relevant to the study are included in the article.

## Supplementary Materials

The following supporting information can be downloaded at: https://www.mdpi.com/article/10.3390/healthcare12242541/s1, Table S1: Risk Analysis—Score assignment: severity (S), occurrence (O) and detectability (D). Table S2: FMECA worksheet: possible failure modes in the preparatory scope. Table S3: FMECA worksheet: possible failure modes in the operational scope.
