# Supplementary material for: Anti-COVID-19 Vaccination in the Italian General Population: Proactive Clinical Risk Analysis Using Failure Mode, Effects, and Criticality Analysis Technique
Source: Healthcare (Basel). 2024 Dec 16;12(24):2541. doi: 10.3390/healthcare12242541 (PMC11675166; doi:10.3390/healthcare12242541)
Supplement: Supplementary file 1 [file healthcare-12-02541-s001.zip › healthcare-3304938-supplementary.pdf]

Supplementary Material

File S1

Balestracci B., Candido G., Federici L., Parretti C., Tartaglia R., Lachman P., Bianco A., La Regina M. Anti-COVID-19 Vaccination in the Italian General Population: Proactive Clinical Risk Analysis Using Failure Mode, Effects, and Criticality Analysis Technique

SUPPLEMENTARY MATERIAL

Supplementary material index

1. **Table S1**: Risk Analysis - Score assignment: severity (S), occurrence (O) and detectability (D).
2. **Table S2**: FMECA worksheet: possible failure modes in the preparatory scope.
3. **Table S3**: FMECA worksheet: possible failure modes in the operational scope.

1. Table S1: Risk Analysis - Score assignment: severity (S), occurrence (O) and detectability (D).

Severity (S)	Occurrence (O)	Detectability (D)
1. No damage	1. Extremely unlikely	1. Almost always detected
2. Minimal damage	2. Low probability	2. Great probability to be detected
3. Moderate damage in the short term	3. Moderate probability, it occasionally occurs	3. Moderate probability to be detected
4. Main damage in the long term	4. Great probability, it repeatedly occurs	4. Low probability to be detected
5. Permanent damage	5. Very high probability, almost inevitable	5. Very low probability to be detected, remote

2. Table S2: FMECA worksheet: possible failure modes in the preparatory scope.

Phase	Activity	Failure Mode	Failure Effects	RPN
Location identification	Identification of vaccination sites according to structural and functional requirements	Structurally unsuitable sites	Failure to identify sites	4
		Functionally unsuitable locations	Failure to identify sites	4
Setting up venues	Preparation of vaccination sites to ensure full and correct functioning.	Lack of involvement of emergency management personnel	Lack/inadequacy of emergency management equipment	10
		Lack of involvement of personal protective equipment (PPE) managers	Lack or inadequate PPE	8
		Lack of adequate cold chain compliance procedures	Loss of doses	15
		Lack of involvement of those responsible for managing consumables and forms (needles, syringes, etc.)	Lack/inadequacy of consumables and/or forms	10
		Lack of common protocols for the vaccination process (hospital and territory)	Increase in adverse events	12
		Inconsistency in the establishment of different General Practitioners (GPs) vaccination sites	Increased risk of adverse events/inefficiency	24
		Lack of involvement of roster management figures	Missing or inadequate shifts in terms of numbers or skills	20
		Lack of/inefficient/delayed recruitment of staff	Missing or inadequate shifts in terms of numbers or skills	20
		Lack of staff training	Staff not (sufficiently) trained	16
		Lack of communication between the people responsible for the different areas of the installation	Failure of the site to function, resulting in delayed or missed administration	15
		Lack of coordination in staging	Failure of the site to function, resulting in delayed or missed administration	15
		Lack of “No Interruption Zone” for vaccine preparation	Administration of an incorrect dose	15
			Errors in vaccine preparation and wastage of vaccine	9
		Lack of collective protection devices (rooms, plexiglass, disinfectants, etc.)	Increased risk of contagion	8
		Lack of involvement of GPs	Lack/inadequacy of GPs emergency management equipment	15
Establishment of information and communications technology (ICT) infrastructure and information technology (IT) hardware and software tools	Set up vaccination booking software	Software unavailability or inadequacy	Reservation management through "poor", non-integrated and poorly controlled tools, resulting in a significant reduction in the quality of managed data and inefficiency of the vaccination process	12
	Set up immunisation registration software	Software unavailability or inadequacy	Reservation management through "poor", non-integrated and poorly controlled tools, resulting in a significant reduction in the quality of data managed and inefficiency of the vaccination process	8
	Set up complementary software (integrations, flows, reports, etc.)	Software unavailability or inadequacy	Inefficiencies in the transfer and communication of managed data	12
	Check/prepare connectivity of vaccination sites	Sites not (adequately) connected	Blocking the start of vaccination activities in sites not (adequately) connected / use of 'poor' tools	10
	Set up computer workstations at vaccination sites	Unavailability/inadequacy of required equipment	Blocked start of vaccination activities at unequipped sites or use of inappropriate equipment	5
	Provide computer training materials for operators	Required content unavailable or inadequate	Compromised quality of training in the use of IT tools and consequent inefficiency of the operators involved	12
Vaccine procurement	Receiving vaccines	Non-reception	Blocking of vaccinations activities	15
		Failure to notify central reservation office of unavailability	Failure to cancel bookings	12
		Late receipt	Loss of doses	12
	Vaccine Control	Broken/altered/expired vials	Loss of doses	3
		Unexpected (lower) number of vials	Slowdown and inefficiency of vaccinations activities	8
		Incorrect temperature on arrival at pharmacy	Loss of doses	5
	Vaccine storage	Storage at non-conforming temperature	Loss of doses	20
		Vial breakage	Loss of doses	4
		Failure to register	Non-compliant inventory	12

	Registering the load into the medicine store	Incorrect registration	Stock levels not in line with actual needs	18
	Updating stock status	Failed to update	Stock not in line with actual data	24
		Incorrect update	Inventory mismatch	24
Vaccination plan design	Establishing periods, days and hours of operation of vaccination centres	Time and place reduction	Slowdown and inefficiency of vaccination activities	8
		Oversize times and locations	Waste of resources	8
	Define the periods, days and hours reserved for certain categories of users	Reducing resources that may be reserved for user types	Slowdown and inefficiency of vaccination activities	8
		Oversizing of resources possibly reserved for user types	Waste of resources	8
	Define the locations, periods, days and hours reserved for the 1st and 2nd dose of the vaccine	Reduction of resources reserved for the two types of services	Slowdown and inefficiency of vaccination activities	8
		Oversizing of resources reserved for the two types of services	Waste of resources	8
		Non-respect of the 2nd dose interval	Reduction in vaccine efficacy	20
	Determining the locations, periods, days and times for the different types of vaccine	Under-resourcing of vaccine types	Slowdown and inefficiency of vaccination activities	8
		Over-allocation of resources reserved for the different vaccine types	Waste of resources	8
	Establish the general calendar of vaccination activities, specifying the days of activity and vaccination sessions at the sites	Failure to set up/incorrectly set up booking agendas, schedules or availability periods, resulting in deviations from the vaccination plan.	Change in the planned timing, pace and volume of vaccination activities (including the possibility of requesting the wrong amount of vaccine)	8
Preparation of reservation agendas	Reservation agenda preparation	Lack or inadequacy of a diary manager	Absence/inadequacy of booking diaries	15
		Inadequate diary management staff in terms of number or competence	Absence/inadequacy of booking diaries	15
Training and education of vaccine operators	Preparing content and materials	Missing or inadequate preparation of required content	Impairment of the quality of training and consequent inefficiency of the operators concerned	12
	Planning the course calendar	Course dates that do not meet the requirements of the vaccination schedule	Slowdown and inefficiency of vaccination activities	16
			Compromised quality of training and consequent inefficiency of operators involved	8
	Delivering the course	Course cancellation/delayed delivery	Compromised quality of training and consequent inefficiency of operators involved	8
		Inadequate course delivery	Compromised quality of training and consequent inefficiency of operators involved	9
	Updating course materials	Failure/delay in updating	Outdated training and increased risk of adverse events/inefficiency	36
		Failure/delay in distribution of updated material	Outdated training and increased risk of adverse events/inefficiency	36

3. Table S3: FMECA worksheet: possible failure modes in the operational scope.

Phase	Activity	Failure Mode	Failure Effects	RPN
Booking Vaccinations	Booking of vaccination appointments based on availability of appointment diaries	Incorrect biographical user identification	Booking made for the wrong user	18
		Incorrect user master data entry	Incorrectly recorded registration status, resulting in problems in re-contacting	12
		Incorrect user category assignment	Inclusion in a schedule that is not in line with the priority established in the vaccination plan for the population concerned	12
		Incorrect vaccine type assignment	Inclusion in a schedule for nother type of vaccine	9
		Booking for wrong dose type (1st or 2nd)	Possible change in vaccination performance	12
		2nd dose booked on non-compliant date	Possible alteration of vaccination performance	15
The process of verifying the availability of essential supplies at vaccination sites and arranging for their replenishment	Checking the availability of consumables (needles, syringes, physiological solution and dye, forms, etc.) and possible procurement	Lack/inadequacy of consumables (needles, syringes, cotton, etc.)	Blocking/delay of vaccination activities at the site(s) concerned	9
		Lack/inadequacy of PPE	Increased biological risk to operator	15
		Lack/inadequacy of forms	Blocked/delayed vaccination activity of participating site(s)	4
	Check the emergency trolley	Lack/inadequacy of emergency management equipment	Increased adverse events for vaccines	15
		Lack/inadequacy of GPs emergency management equipment	Increased adverse events for vaccines	15
Creation of vaccination teams	Manage the rostering of doctors on the vaccination teams, including the identification of on-call substitutes	Failure to identify the medical components	Inability to carry out the vaccination	12
		Failure to identify medical components substitutes	Impossibility to carry out the vaccination session or reduction of the session in case of absence of the holder(s)	8
		Incomplete identification of medical component and/or substitutes	Reduction of the vaccination session in the absence of the vaccinators and/or their deputies	6
	Manage the rostering of nurses, medical assistants and social-health workers on the vaccination teams, including the identification of on-call substitutes	Failure to identify care and social/health components and substitutes	Inability to carry out the vaccination	10
		No identification of care and social/health component and substitutes	Inability to administer the vaccine	10
		Incomplete identification of nursing and social-medical component and/or substitutes	Reduction of the vaccination session in the absence of the vaccinators and/or their deputies	6
	Manage the rostering of administrative staff on the vaccination teams, including the identification of on-call substitutes	Administrative component not identified	Inability to carry out the vaccination or reduction of the vaccination in case of absence of the owners	8
		No identification of substitutes for the administrative component	Impossibility to carry out the vaccination session or reduction of the session in case of absence of the holder(s)	8
		Incomplete identification of administrative component and/or substitutes	Reduction of the vaccination session in the absence of the vaccinators and/or their deputies	6
Availability of the list of vaccines during the vaccination session	Obtaining and checking the list of vaccinees for each planned vaccination session	Failure to acquire the list	Blocked/delayed vaccination activity at site(s)/location(s)	15
		Failure to validate the list	Blocked/delayed vaccination activities at the site(s)/location(s) involved	15
		Incorrect list availability	Blocked/delayed vaccination activities at the site(s)/location(s) involved	15
Requests for vaccines from the pharmacy	Requesting the hospital pharmacy to prepare the vaccines in the correct quantities for the different sites and planned vaccination sessions on a specific date	Failure to send request to pharmacy	Missed/delayed vaccination	27
		Request sent to pharmacy late	Delayed vaccination	15
		Wrong dose number request (lower)	Missed/delayed vaccination	12
		Wrong dose number request (higher)	Need to enroll additional vaccinees / need to store excess vials at an appropriate and controlled temperature	5
Managing the transfer of vaccines from the pharmacy to the vaccination sites	Preparing deliveries according to the planned procedures and recording unloading from the pharmaceutical store with updating of stock status Subsequent delivery to the vaccination sites	Failure to send vaccine doses	Missed/delayed vaccination	5
		Late delivery of vaccine doses	Delayed vaccination	8
		Delivery of wrong number of doses (lower)	Missed/delayed vaccination	5
		Wrong number of doses delivered (higher)	Need to enroll additional vaccinees / need to store excess vials at an appropriate and controlled temperature	5
		Failure/inadequate maintenance of cold chain	Loss of doses	15

Implementation of vaccination activities	Management of vaccinees on site, recognition and delivery of forms	Assemblies	Increased risk of infection	12
		Missing/incorrect recognition	Administration of vaccines to persons below the age limit specified in the product information	6
			Administration of correct vaccine to wrong user / Administration of incorrect vaccine to correct user	24
		Missing/inadequate delivery of forms	Deceleration of vaccination activity	6
	Data collection and form completion	Incomplete/erroneous/missing data collection and completion of forms	Increased risk of adverse reactions	24
		Failure to sign consent	Unauthorised administration	10
	Vaccine administration	Change of vaccine type	Administration of wrong vaccine	24
			Inappropriate vaccine administration by user type	24
		Injection of the vaccine	Musculoskeletal injury	48
			Subcutaneous rather than intramuscular administration	36
		Change of appointment	Failure to schedule a 2nd dose for 1st dose subjects	6
			Scheduling a 3rd dose for 2nd dose subjects	6
		Vaccine switch between 1st and 2nd dose	Violation of the principle of non-interchangeability	12
	Vaccination registration	Vaccination registered to wrong user	Change in vaccination record	6
		Failure to register vaccination	Change in vaccination record	6
	Preparation of reserve list	Missing reserve list	Unused doses	5
		Insufficient reserve list	Unused doses	5
		List of ineligible subjects	Failure to respect the priorities of the vaccination plan	15
	Contact with reservists	Reserves not contactable	Unused doses	12
		Reserves not available	Unused doses	12
	Management of session-related adverse events	Failure/inadequate management of immediate adverse reactions	Death/serious harm	6
	Prevention of adverse reactions during the session	Failure/inadequate prevention of serious allergic and non-allergic adverse reactions (1st dose)	Death/Serious injury	60
		Failure/inadequate prevention of serious allergic and non-allergic adverse reactions (2nd dose)	Death/Serious injury	10
Management of vaccination documentation	Failure/incomplete archiving of medical history and individual consent forms	Lack of/incomplete traceability of vaccination activity	Missing/incomplete traceability of vaccination activity; lack of consent for 2nd dose	5
	Failure/incomplete archiving of session record sheet	Lack of consent for 2nd dose	Missing/incomplete traceability of vaccination activity; lack of consent for 2nd dose	5
Reporting of vaccination activities to the region	Reporting on the activities carried out during the vaccination sessions in accordance with the established procedures and forwarding the data processed in this way to the competent authorities through the established channels	Presence of inconsistent or incomplete data	Logical, formal or substantive errors in procedures	6
		Non-recording of vaccinations	Incomplete flow compared to actual vaccination activity	6
